# Disaster preparedness and resilience at household level in Yangon, Myanmar

**DOI:** 10.1007/s11069-022-05226-w

**Published:** 2022-02-11

**Authors:** Sophie-Bo Heinkel, Benni Thiebes, Zin Mar Than, Toe Aung, Tin Tin Kyi, Win Lei Mar, Saw Sandar Oo, Christian Miller, Marlene Willkomm, Win Maung, Zin Nwe Myint, Khin Khin Soe, Regine Spohner, Frauke Kraas

**Affiliations:** 1 Institute of Geography, University of Cologne, Albertus-Magnus-Platz, 50932 Cologne, Germany; 2 German Committee for Disaster Reduction (DKKV), Kaiser-Friedrich-Straße 13, 53113 Bonn, Germany; 3 Yangon City Development Committee (YCDC), Yangon City Hall, No. 420/450, Mahabandoola Road, Kyauktada Township, Yangon, Myanmar; 4 Cologne Fire Department, Institute for Security Science and Rescue Technology (ISR), Scheibenstraße 13, 50737 Cologne, Germany; 5 Flood Protection Centre of the Municipal Drainage Operation of the City of Cologne (StEB Köln), Ostmerheimer Straße 555, 51109 Cologne, Germany; 6 Myanmar Environment Institute (MEI), C-106, Delta Plaza Bldg., Bahan Township, Yangon, Myanmar; 7 Department of Geography, University of Yangon, Kamayut Township, Yangon, Myanmar

**Keywords:** Risk management, Disaster preparedness, Societal resilience, Cyclone, Earthquake, Flooding, Yangon, Myanmar

## Abstract

Resilience has become important in disaster preparedness and response. Unfortunately, little is known about resilience at the household level. This study presents the results of a survey into individual and household level preparedness to disaster events in Yangon, Myanmar, which is prone to natural disasters such as tropical cyclones, flooding, and earthquakes. The study aimed to understand societal resilience and to provide information that could be used to develop a holistic framework. In four different Yangon townships, 440 households were interviewed. The results of the survey indicate how risk preparedness could be improved by specific measures related to the following five factors: (1) increasing the general public’s knowledge of first aid and its role in preparedness; (2) improving mobile phone infrastructure and capacity building in its usage so that it can be used for communication during disasters, along with building up a redundant communication structure; (3) better use and organisation of volunteer potential; (4) more specific involvement of religious and public buildings for disaster response; and (5) developing specific measures for improving preparedness in urban areas, where the population often has reduced capacities for coping with food supply insufficiencies due to the high and immediate availability of food, shops and goods in regular times. The findings of this survey have led to specific recommendations for Yangon. The identified measures represent a first step in developing a more general framework. Future research could investigate the transferability of these measures to other areas and thus their suitability as a basis for a framework.

## Introduction

In recent years, the term resilience has been used increasingly in many fields of society and in the science and practitioners’ communities dealing with disaster risk reduction, sustainable development, and climate change adaptation (Folke et al. 2002; Alexander 2013; Etinay et al. 2018; Leal Filho et al. 2018; Woodruff et al. 2018; Elmqvist et al. 2019). However, the understanding of resilience varies and different aspects of stability and flexibility are emphasized. This is not surprising given the development of resilience theories.

Resilience as a concept originated in the early nineteenth century in the field of material science, where it was used to describe the ability of materials to absorb energy without undergoing permanent deformation (Sudmeier-Rieux 2014). In the 1960s and 1970s, psychological research used the term resilience to describe the ability of humans to recover from traumatic events (e.g. Nutting and Norris 1968). Antonovsky’s concept of salutogenesis defines resilience as driving force of a sense of coherence, which he describes as essential for staying healthy (Antonovsky 1979, 1996). This definition of resilience found its way to health geography in order to understand impacts of landscape constructions on human health (Gesler 1992; Kearns and Gesler 1998). Concepts of resilience gained substantial prominence in the field of ecosystem research and were refined iteratively and subsequently adopted in other fields. There, initially, resilience was used to describe the ability of ecosystems to cope with and withstand external stress, e.g. when a coral reef can withstand a temperature rise and not die off. Inherently, this refers to the ability to recover and “bounce back” after disturbances. This understanding of resilience, termed engineering resilience (Holling 1973; Hollnagel et al. 2006), thus referred to stability, resistance, and rigidness. Acknowledging that systems can have multiple equilibriums, ecological resilience is based on the understanding that recovery does not necessarily mean returning to the initial condition; it is more likely to lead to new quasi-stable system states (“bouncing forward”) (Hollnagel et al. 2006; Hollnagel 2013). This concept of resilience was later extended to socio-ecological systems, providing a conceptual framework of nested systems interacting with each other across scales (panarchy) (Holling and Gunderson 2002). A strong focus is put on the transformations and interactions of systems across various scales that grow, mature, collapse, and renew. Thus, resilience is understood more as a process of transformation and adaptation, also facilitated by learning and remembering from past transformations.

Today, resilience has become the guiding principle and explicit goal of multiple international frameworks, e.g. the Sustainable Development Goals (United Nations 2015a), the Paris Climate Agreement on Climate Change (United Nations 2015b), the Sendai Framework for Disaster Risk Reduction 2015–2030 (UNDRR 2015), and the New Urban Agenda (UN Habitat 2016). The definitions of resilience between the frameworks differ slightly, however, a recent report by UNDP (2020) attempts to streamline them to foster societal resilience building. That report defines resilience as “*the ability of individuals, households, communities, cities, institutions, systems and societies to prevent, resist, absorb, adapt, respond and recover positively, efficiently and effectively when faced with a wide range of risks, while maintaining an acceptable level of functioning without compromising long-term prospects for sustainable development, peace and security, human rights and well-being for all*” (UNDP 2020, p. 7). Resilience is, accordingly, defined by a set of capacities and resources that are crucial to cope with, withstand, and bounce back from adverse events and shocks, i.e. the (i) absorptive, (ii) adaptive, (iii) anticipative, (iv) preventive, and (v) transformative capacities. By adopting this understanding of resilience, we aim to apply a very integrative and holistic understanding of resilience. Resilience is thereby very much understood as a framework that emphasizes the ability of individuals to act under various shock and stress events. However, this does not relate to a quantification of resilience with respect to the capacities which are rather regarded as different qualitative aspects of personal actionability. In addition, the understanding of resilience explicitly widens the focus from pure safety focus system stability to long-term stability reflecting sustainable development.

During the initial period of extended natural disasters, it can be assumed that emergency medical care and public emergency response services are concentrated on focal points and that many affected areas cannot be supplied initially. Therefore, individual resilience on the household level and community resilience contribute substantially to mitigation in the early stages of disasters (Donahue et al. 2014; UNOCHA 2017a). People can increase their ability to cope with, adapt to, and recover from hazard impacts by preparedness (Paton 2006; Paton et al. 2013). However, studies revealed that the level of individual preparedness is influenced by personal perceptions of individual risks and circumstances (Paton et al. 2013). Therefore, informing individuals about risks is not enough; preparation awareness must be promoted, too. Resilience on a household level can be improved on the level of preparedness for medical emergencies and for supply insufficiencies (water and food, energy, information, and communication), and with knowledge about behaviour and evacuation situations. Community-based resilience can be improved through experience and correct behaviour.

Community resilience emerges from a set of networked adaptive capacities with dynamic trajectories having potential to maintain function during and adapting in the aftermath of disasters (Norris et al. 2008). Despite the increasing importance of the resilience concept, there is not yet a common understanding of how resilience can be quantified and practically applied to different systems (e.g. households, social groups, cities, regions, countries). With this study on household and personal preparedness with respect to disaster events, we contribute to the understanding of how, particularly, the anticipative and preventive capacities could be analysed.

This study presents an analysis of individual and household level preparedness to disaster events. Using the responses to questionnaires, we elaborate on the current state of households with respect to their individual preparedness and their anticipative, preventive and adaptive capacities to react to disaster events caused by natural hazards. In four different townships of Yangon, Myanmar, 440 households were interviewed, focusing on important aspects of resilience at the household level and opportunities to improve knowledge about anticipative and preventive capacities, such as abilities to react early to natural hazards and to reduce the risk of impacts. The data were disaggregated by township, education, sex, age, and occupation. The study contributes to understanding societal resilience and providing information towards developing a holistic framework. We also derive recommendations for the local administration to improve societal resilience and capacities for absorption of and adaptation to natural hazards.

## Risk profile of Yangon

Due to its geographical location, Myanmar is prone to natural disasters such as tropical cyclones, flooding, and earthquakes. The Sagaing fault runs through the centre of the country and is known for its seismic activity and for frequently causing landfalls and earthquakes of various magnitudes (Hla Hla Aung 2009, Thein et al. 2009, Soe Thura Tun and Watkinson 2017). Major cities such as Yangon, Mandalay, and the country’s capital Nay Pyi Taw are located on this fault, others such as Taunggyi and Mawlamyine are situated on side fault lines (Kyaukkyan fault and Bilin fault) (Kraas et al. 2017). Myanmar also is exposed to tropical cyclones, which develop in front of the coastal areas through the mix of hot air masses from landmasses and colder oceanic air masses. They mainly occur in the pre- or post-monsoon seasons in April/May and October/November. Tropical cyclones reach the coastal areas and often run through the country at high speed. Sedimentary analysis shows that the coastal shoreline has shifted during tropical cyclones and tsunamis between 15 and 70 m landwards (Brill et al. 2019). Within the last 15 years, Myanmar has been affected by at least four severe tropical cyclones. Cyclone Nargis in May 2008 cost about 138,000 victims (Kraas 2009; Kraas et al. 2017). Cyclone Giri in October 2010, cyclone Komen in August 2015, and cyclone Maarutha in May 2017 caused devastating damage (Brakenridge et al. 2017; UNOCHA 2017b). Cyclone Nargis, in particular, affected the Ayeyarwady Delta and the megacity Yangon and left vast flooded areas around the city (Heinkel et al. 2021).

**Fig. 1:**
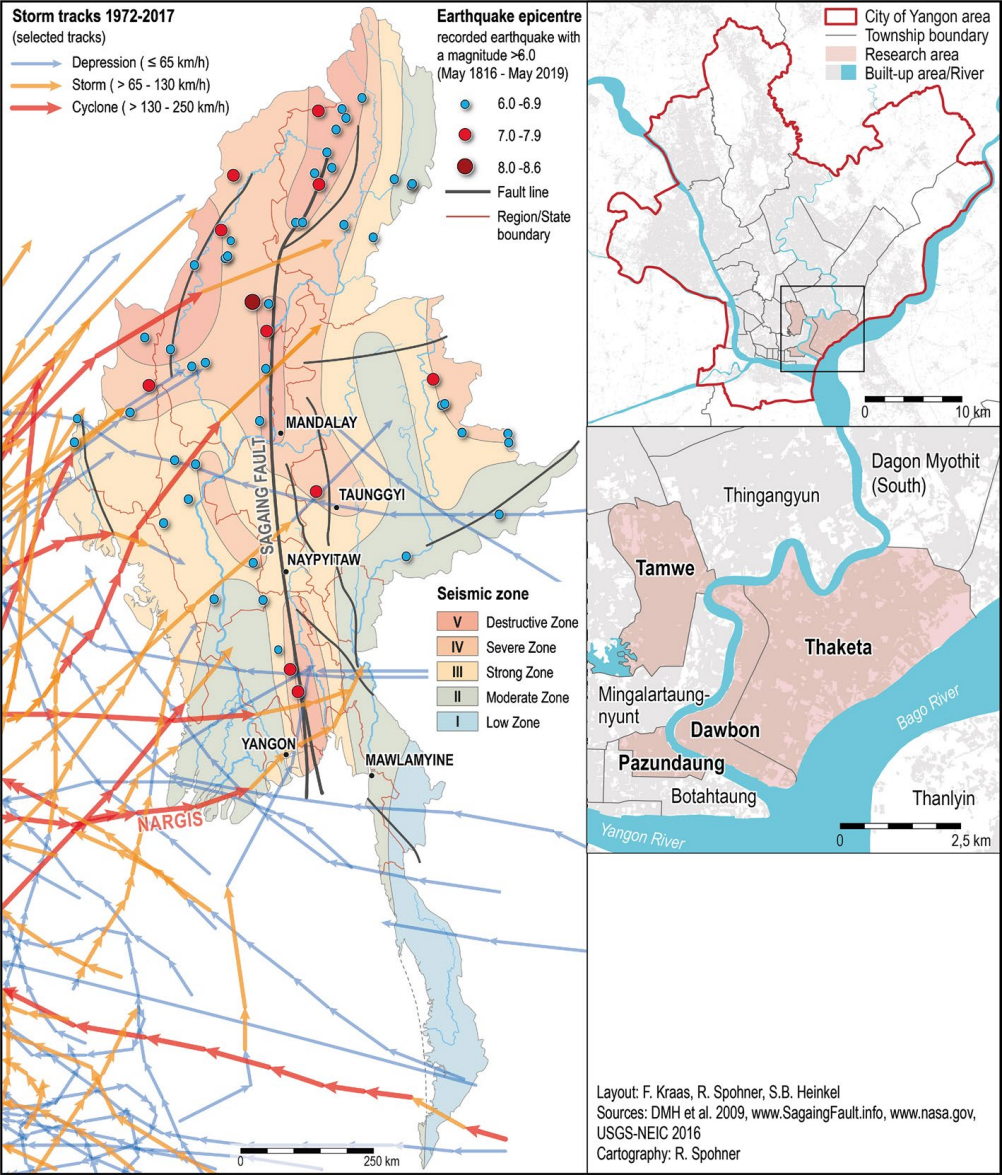
**a** Myanmar risk profile and **b** surveyed Yangon townships

Yangon is surrounded by three rivers and creeks, where flooding occurs from either high tide or heavy rainfalls during the monsoon season or tropical storms in the pre- and post-monsoon seasons. The megacity (UN DESA 2015; Kraas et al. 2017, 2019), with its more than 5 million inhabitants, is growing rapidly and therefore exposed to human-made hazards such as flooding by blocked drainages due to rapid urbanization and uncontrolled settlements (Zin Mar Than et al. 2020; Hartanto and Rachmawati 2017, Rachmawati and Budarti 2017). Weak solid waste management and uncontrolled plastic waste disposal in the open drainage systems more often result in blockages of the sewage system and cause flooding of rain and sewage water. This is problematic since the flooded water might not only block transport channels but also poses a health threat, especially for children, due to contamination with pathogens causing diseases (Heinkel et al. 2021) (Fig. 1).

The CoViD-19 pandemic has also had a strong impact on the whole country. During the lockdowns in 2020, large business districts in Yangon were closed and the population was ordered not to cross township borders. Political decisions set a country-wide “stay-at-home” campaign to contain CoViD-19 outbreaks and not overload the health system. While this strategy was successful in preventing disastrous consequences of the pandemic, these measures severely weakened the economic situation of the majority of the people in Yangon and Myanmar. Private protection against natural hazards or losses of (economic) livelihoods, such as financial reserves, only exist in richer communities and those with more international citizens and expats, such as in Kamaryut and Bahan Townships, while the majority of Yangon’s citizens does not have an insurance against disasters, such as flooding.

An institutional disaster management was initialized after cyclone Nargis in 2009, when the government adopted the Myanmar Action Plan for Disaster Risk Reduction (RRD 2009). This document was last updated in 2017 (NDMC 2017). It outlines actions for increasing general disaster preparedness (NDMC 2017). The national Department of Disaster Management (DDM) requires that resource disaster assessment set priorities in national disaster risk management (DRM). Furthermore, potentials for improving disaster management must be assessed. Even though these strategic frameworks underline the priority placed on disaster preparedness for increasing disaster resilience, the institutional disaster preparedness in Yangon’s townships remains weak (Zin Mar Than et al. 2020), e.g. risk communication and training for providing disaster education are not well established. The institutions lack the flexibility to adapt to disastrous situations and have limited capacity to absorb shocks. Little effort is made to prepare the population. In 2019, the NDMC revealed the National Earthquake Preparedness and Response Plan 2019 (NDMC and UNDP 2019), which was immediately adapted to Yangon. Since then, national and regional television stations have been communicating risks about earthquakes and showing target-group-oriented earthquake preparedness films.

## Methods

### Household survey

In February 2020, imminently before the CoViD-19 pandemic, a household survey on private disaster preparedness and the willingness to volunteer in disaster preparedness was conducted in the City of Yangon. Four townships, Pazundaung (48,455 inhabitants), Tamwe (165,313 inhabitants), Dawbon (75,325 inhabitants), and Thaketa (220,556 inhabitants), were selected for the survey due to their geographical location between or nearby rivers. The townships are prone to flooding due to high precipitation during the monsoon season and river floods or to high-tide during and after tropical cyclones and flooding by blocked drainages. Pazundaung and parts of Dawbon developed within the colonial phase of urban development from 1852 to 1948 and are, therefore, among the oldest parts of the city. Today they are part of the inner urban townships. Pazundaung and Tamwe are characterised by a high building density with less green space (Kraas et al. 2010; Kraas 2019) (Table 1). The main parts of Dawbon and Thaketa developed later, after independence in 1948 when the city expanded due to migration and urbanisation processes. The main increase in the urban population resulted in the so-called new towns. Further buildings have emerged since 1966, during the planned economy phase after the military coup in 1962. Today, Dawbon and Thaketa are characterised by densely built-up areas with regular tree population and trees along the roads (Kraas et al. 2010). Pazundaung and Tamwe have higher population densities and a larger elder population than Dawbon and Thaketa. More professional and clerical support workers can be found in inner urban townships. Dawbon and Thaketa have more craft and related sales workers (Table 1).

**Table 1:** Demographic indicators of the sample sites. Source: DoP (Department of Population), MoLIP (Ministry of Labour, Immigration and Population) 2017a, 2017b, 2017c, 2017d

Township	Pazundaung	Tamwe	Dawbon	Thaketa
Area in km^2^	1.0	4.4	3.8	12.8
No. of wards	10	20	14	19
Population size	48,455	165,313	75,325	220,556
Population density (persons/km^2^)	47,894.0	37,374.0	19,738.3	17,257.9
Mean household size (persons)	4.4	4.4	5.0	4.7
Population age	0–14: 16.9%; 15–64: 74.9%; > 65: 8.2%	0–14: 17.5%; 15–64: 75.0%; > 65: 7.5%	0–14: 23.4%; 15–64: 71.4%; > 65: 5.2%	0–14: 20.2%; 15–64: 73.4%; > 65: 6.4%
Main businesses (with shares > 10%)	30.5% service and sales workers; 12.0% craft and related trade workers; 11.0% professionals; 10.6% clerical support workers	32.5% service and sales workers; 12.9% craft and related trade workers; 10.4% professionals; 10.0% clerical support workers	38.5% service and sales workers; 22.7% craft and related trade workers	30.4% service and sales workers; 27.9% craft and related trade workers

### Sampling procedure

In total, 440 households were selected through a stratified sampling method. Township and ward administrative levels served as strata, and thus 110 interviews per township and, more detailed, 11 interviews per randomly selected ward were collected. The quantitative questionnaire contained eight sections with a mix of open and closed questions about (i) risk response behaviour, (ii) information and communication behaviour, (iii) food and water stockpiling, (iv) alternative energy sources, (v) used modes of transportation, (vi) preparedness in health care, (vii) safety and security concerns, (viii) perception and preparedness concerning the new Corona virus, and (ix) cooperation potential of the civil society. The sample was statistically analysed using IBM SPSS. Due to the lack of normal distribution, nonparametric tests, such as the Kruskal–Wallis-test and the Mann–Whitney-test, were conducted and the Spearman range-correlated coefficient was used for the statistical analysis.

### Measurement of disaster preparedness at the household level

Individual disaster preparedness is a key aspect of resilience in low- and middle-income countries (Hoffmann and Muttarak 2017), where rescue and disaster management systems often do not have sufficient capacities for immediate and efficient disaster response. International assistance can usually mobilize funds and response measures within the first 72 h after the disaster (Russell et al. 1995; Peters and Kraas 2014; UNOCHA 2017a). The local population, therefore, needs to cope with supply insufficiencies of critical infrastructures, such as medical emergencies, within the first three days after a disaster. The availability of resources such as food, water, medical provisions and first-aid kits, and electricity alternatives for this time period saves lives during and after disasters (Petit et al. 2011). Thus, we defined stockpiling of food and drinking water and the availability of alternative power sources for cooking, lighting, and alternative access to information as key indicators of individual disaster preparedness and societal resilience.

Key prerequisites of anticipating, preventing and adapting capacities during disasters are also risk awareness and adequate knowledge about (natural) hazards and the right behaviour before, during, and after a disaster. This knowledge can be provided by education and experience. In fact, studies have pointed out the positive impact on disaster preparedness of formal education, such as in nursery schools, schools and universities (Hoffmann and Muttarak 2017), and informal education, such as community-based methods to reduce disaster risks (Allen 2006; Maskrey 2011). Education provides knowledge on disasters and improves the ability to imagine and understand natural disasters. Furthermore, an increased flexibility in acting are educational outcomes with positive effects on preparedness and resilience (Hoffmann and Muttarak 2017). Indirect educational effects also increase the household resilience due to the associations between education and income, housing, and social and mental well-being (Birkmann et al. 2016; Hoffmann and Muttarak 2017).

Several studies have also shown that experience of disasters increases the chances of better preparedness (Guo and Li 2016; Roder et al. 2016; Deng et al. 2017; Hoffmann and Muttarak 2017). These case studies have demonstrated that disaster experience is highly related to the geographical location of a person’s housing. However, it is still unclear at which scale these differences in knowledge and preparedness appear. Thus, we investigated intraurban similarities and differences in the disaster knowledge and behaviour within different townships in Yangon.

Social support during disasters is a further central part of local disaster resilience. Social support includes daily exchange and social networks with others (Scherer and Cho 2003; Djalante et al. 2020) and the acquisition of volunteers. Intraurban differences might appear in a geographical but also in social environments. This study also assessed the township inhabitants’ social willingness to voluntary help before, during, and after disasters.

## Results

**Fig. 2:**
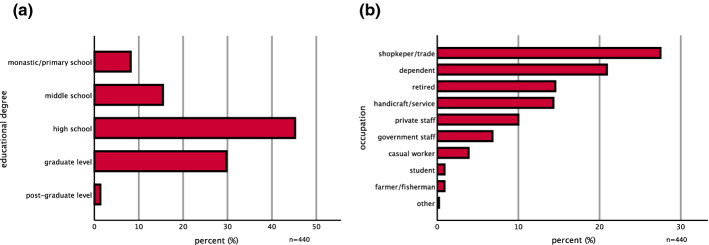
**a** Educational level; **b** occupation

The sample consisted of 63.0% male and 36.8% female participants with an average age of 52.7 years. The households consisted of 5.0 persons (mean) with a range of 1 to 25 persons per household. The majority had a high school degree (45.2%) or graduate level degree (29.8%), a minority attended only middle schools (15.5%) or primary or monastic schools (8.2%) (Fig. 2a). The main income sources were either shop-keeping and small trade (27.5%) or being dependent on other income (20.9%), or retired (14.5%). Other income sources were handcraft and services (14.3%), private employment (10.0%), and others (12.8%; government employment, casual workers, farmers/fishermen, students) (Fig. 2b).

### Coping with supply insufficiencies in urban areas

**Fig. 3:**
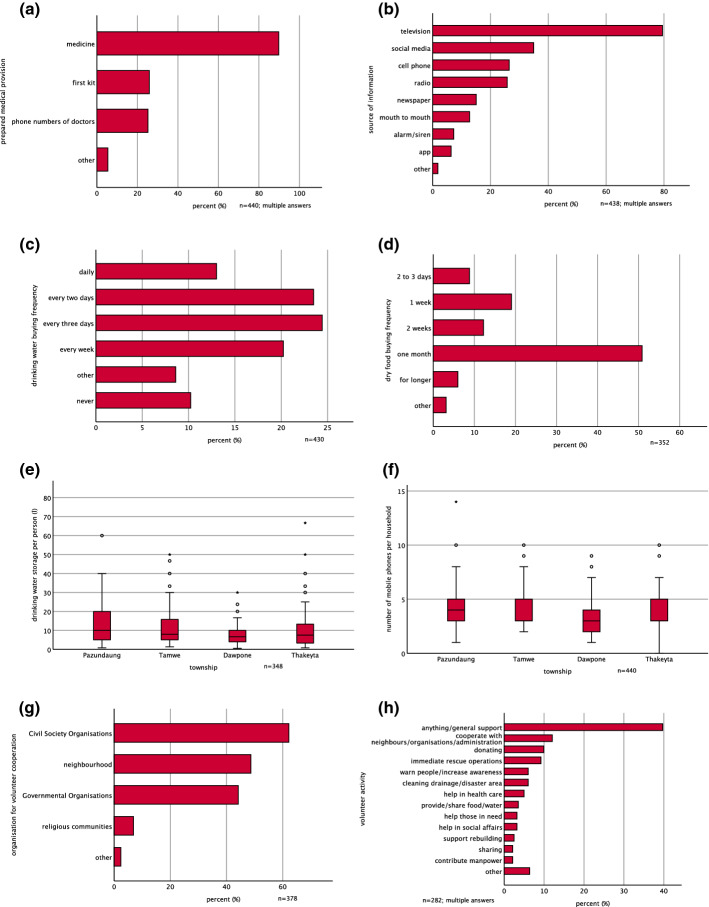
**a** Prepared medical provision; **b** information sources; **c** drinking water buying frequency; **d** dry food buying frequency; **e** drinking water storage frequency in townships; **f** mobile phones in townships **g** volunteer organisations; **h** voluntary tasks

Most respondents (71.6%), independently from age or sex, stated that they had prepared medical provision for the case of a medical emergency. However, only 25.9% of those had first-aid kits at home or separately noted contact numbers of doctors or health stations (25.2%). Most people see medical provision only in the preparation of necessary medicine for family members (89.9%). 28.4% of the households were not prepared at all (Fig. 3a).

In the investigated townships, it is common to use bottled drinking water from barrels ( mean 63.1%) in water dispensers (DoP, MoLIP 2017a, 2017b, 2017c, 2017d). These barrels contain 20 L of water and are sold and delivered by mobile water vendors or bought from shops and supermarkets. This trend was reflected in the survey, detecting 80.3% of the respondents using drinking water from bottled water. Only 10.7% of the respondents had a small-scaled drinking water supply or opportunities for water purification and were not dependent on these water vendors. The total median number of stored litres of drinking water per household member was 8 L, including a high range of 1.0–95.0 L per person. However, there was a significant difference (*p*-value: 0.02) between the townships of Pazundaung (median 10.0 L) and Dawbon (median 6.7 L) (Fig. 3e). In total, 60.9% of the households needed to buy water at least every 3 days (Fig. 3c).

That buying frequency is high compared to the buying frequency for dry food. Only 8.8% of the respondents stored dry food for 2–3 days, 19.0% had dry food for one week at home and 12.2% for two weeks. 56.9% stored dry food for one month or longer (Fig. 3d). There was no significant difference in stockpiling of food between townships, education, sex, or occupation, while those who stockpiled food were significantly older than those who did not (*p* < 0.01). Interestingly, 18.9% did not store dry food at all. Besides a shortage of money (15.2%), many of the reasons for the lack of stockpiling are based on the urban settlement characteristics: people said that shops are nearby (16.7%) and the household buys food daily in daily neighbourhood markets (19.9%). Consequently, they saw no need (4.5%) for stockpiling dry food. Also, small and single household sizes (7.6%) and less storing space (4.5%) prevented people from stockpiling. The wide variety and availability of street food has changed peoples’ cooking behaviour. Especially in shared living spaces, e.g. for male workers, people did not cook at home at all (7.6%) and, therefore, did not store any food. Other respondents thought that stockpiling food would be unhealthy (9.1%) or they were afraid of vermin infestation or expiring food (4.5%). Finally, 6.1% just did not want to store dry food.

People were asked whether they had alternative cooking facilities and light sources in case of short- and medium-term power cut-offs, which occur relatively frequently. The majority (87.0%) had alternative cooking facilities for times when electricity was interrupted. The main alternative cooking sources were charcoal (42.7%) and gas (42.3%); firewood was less often mentioned (1.8%). Additionally, 88.4% had alternative light sources. The main light sources were LED lights (24.5%). Besides this source, a high variety of alternative light sources were mentioned such as battery-driven torches (11.8%), candles (10.7%) or emergency lights (9.5%), chargeable lights (8.6%), solar lights (1.4%), flashlights (0.9%), or even generators (9.3%) or inverters (6.8%). Explicit knowledge about how long the light alternatives would last could not be assessed. Estimations of how long the LED lights, candles or battery-driven lighting alternatives would last diverged greatly. However, 50% (median) of the respondents said that their light alternatives would last for less than half a day (0.4 days). 25% of the sample had light alternatives for 2 days or less.

The interviewees were also asked whether they had alternative sources of information, for the case when, e.g. television or internet were disrupted. The majority (79.5%) relied on television as a source of information. However, because they need continuous electricity, alternative information sources become relevant during power cuts. 41.3% also used social media and apps mainly from mobile phones (26.5%) to get information. 25.8% mentioned the radio as an information source in case of disasters. Fewer people relied on newspapers (15.1%), mouth-to-mouth information (12.8%) and alarms or sirens (7.3%) (Fig. 3b).

In general, only one of three households had a radio (median 0; mean 0.4), which excludes radios as a household and area covering source of information in case of disasters. In contrast, the median number of mobile phones per household was three; only two out of 440 households had no mobile phone. There is a significant difference (*p*-value: 0.01) in numbers of mobile phones per household between Dawbon and the townships Pazundaung and Tamwe (Fig. 3f). Households in the inner urban townships tended to have slightly more mobile phones per household than households in the new towns. Using mobile phones for risk and crisis communication would mean that the access to knowledge is ensured in 95% of the households. Power banks (30.7%) to charge phones are more common than any other alternative power supply. This means that most people are prepared for short-term power-cuts but not for longer electric power supply disruptions.

### Knowledge of behaviour during natural hazards and evacuation situations

The household survey in February 2020 revealed that 91.1% of Yangon’s population, independently from age or sex, had already experienced a disaster. Significant correlations (*p* < 0.01) exist between the different townships and the experiences of cyclones and flooding. While almost all participants in the townships of Tamwe, Dawbon, and Thaketa (90.0–91.8%) had already experienced a cyclone, in Pazundaung 73.6% said they had experienced such an event. We assume that in Tamwe the high number of people who have experienced a cyclone is due to the fact that, within the last years after Nargis, there was an influx of migrants from the Ayeyarwady Delta to Yangon townships, including Tamwe (Pattison et al. 2016; O’Connor 2020). The difference in experiences between Pazundaung and Dawbon and Thaketa is due to the age structure of the survey sample. Even though Dawbon and Thaketa have a younger population than Pazundaung and Tamwe (DoP, MoLIP 2017a, 2017b, 2017c, 2017d), one which might not have experienced cyclones, this age distribution is not reflected in the survey data. In fact, Dawbon and Thaketa have a higher median age (53; 54 years) than Pazundaung (50 years). Younger people might not know or remember if their households had experienced specific extreme events, posing a response bias here.

With respect to flooding experiences, the townships of Dawbon (34.5%) and Thaketa (28.1%) appeared as more exposed than the townships of Tamwe (19.1%) and Pazundaung (10.1%). Only 15.9% of the respondents noted that they had experiences with earthquakes.

Answers to the open questions about behaviour in case of these natural hazards were diverse and, in many cases, contradictive. In case of a tropical cyclone, 34.1% of the households would move to a shelter, 25.3% would stay at home. Both forms of behaviour were mentioned in all townships and by people at all educational levels. Seven per cent, mainly from Dawbon and those with high school and graduate degrees, would open the door and windows, probably because, during past cyclones, people were instructed by authorities to do so. However, 2.7% would do the opposite. A small number of people would increase the house’s resistance against cyclones (3.8%) and/or repair damages from the storm immediately (2.4%). Other answers referred to helping (6.5%) or warning (3.0%) family, friends, and neighbours. Also, cooperation with neighbours, organisations, and administration (4.6%) was among the coping and disaster response strategies.

The answers to questions about flooding were less contradictive. 34.0% would evacuate to higher places or lift things up to higher floors. This answer came mainly from participants in Dawbon and Thaketa and also from people with higher educational degrees (high school and graduate school). A clear focus in all townships was on helping family, friends, and neighbours (19.3%) and also on cooperation with neighbours and organisations (7.5%). This factor, however, was only mentioned by people with higher educational degrees. Many people were aware that one of the human-made reasons for flooding are blocked drainages; thus 14.7% would clean or check the drainage system. Fewer people in Pazundaung referred to this aspect but more in Tamwe, Dawbon, and Thaketa. Other important strategies were ‘keeping important things for evacuation’ (6.1%), ‘building water barriers and sandbags’ (3.7%), and the ‘construction of boats and life jackets from plastic bottles’ (1.9%).

In case of an earthquake, the majority of respondents would go outside to open spaces (37.1%) or move to unspecified safe places or a shelter (25.7%). In contrast, 14.7% would stay at home. Only 9.3% of the participants, mainly those from Pazundaung and Tamwe and with higher educational degrees, would stay under furniture. 7.7% would also warn or help others.

In all cases of natural hazards, only few people would follow the news and official warnings or weather forecasts (cyclone: 2.4%; flooding: 0.5%; earthquake: 1.4%). Also, the saving of important documents seemed to be neglected before and during disasters (1.6%; 0.0%; 1.4%) (Zin Mar Than et al. forthcoming). While the answers to questions concerning flooding seemed to be more directed and solution focused, the behaviour during tropical cyclones and earthquakes seemed to be highly divers and partly contradictive. This result indicates a gap of knowledge concerning appropriate behaviour during certain natural hazards. Most of the answers came from participants in Dawbon (26–28%). Participants in Thaketa had diverse strategies to reduce flooding disaster risk. Those two townships have comparable, large riverbank areas and are impacted by high tide flooding from time to time. It seems that the geographical location of the townships, and thus previous experience with flooding, has an impact on inhabitants’ behaviour during disasters such as flooding.

In the survey, people were asked for the five most important things to carry in case of evacuation. Many respondents were aware of the main important belongings, such as (i) important documents (122.9%),^1^ (ii) money and jewellery, gold and valuables (68.8%), (iii) food and water (49.7%), (iv) clothes (27.0%), and (v) medicine (19.4%). Also, mobile phones (14.2%) as alternative sources of information with charged power banks and light sources (13.7%) were ranked as important belongings. Battery-driven radios (0.3%) were less often mentioned. For the important documents, 227 respondents specified their answers. The mostly mentioned documents were: (i) National Registration Card or identity card (30.8%), (ii) the household census list (12.8%), (iii) bank account documents (4.7%), and (iv) house ownership contract (3.4%). Other documents were less often mentioned but were also very important for preventing the loss of personal property. The extracted list of documents provides a good overview about the perception of important documents. Family photos, owner books, land grant, contracts and medical records—as recommended for Yangon (YCDC et al. 2020)—and vaccination certificates, membership and contribution books of associations or clubs (BBK 2017) should be added to national and international recommendations in lists of personal documents.

### Potentials for better involvement of volunteers for a disaster resilient society

Most respondents (89.3%) were willing to voluntarily support institutions and the general public in disaster management. However, only 3.9% were already involved in voluntary disaster prevention activities, this group of people consists of significantly more men (*p* < 0.01) and was significantly older than those who were willing to collaborate but not yet involved in activities (*p* = 0.03). These respondents came mainly from Dawbon and Thaketa. As mentioned, many people are concerned about helping family, friends, and neighbours in emergency cases. This civil society attitude of caring provides a high potential for increasing and improving current disaster prevention on a cost-efficient and voluntary basis. People wanted to cooperate with civil society organisations (62.2%), the neighbourhood (48.7%), or with governmental administrative units (44.2%). Surprisingly, only 6.9% would like to cooperate with religious communities in terms of voluntary actions of disaster prevention (Fig. 3g). Religious institutions and religious buildings, however, play an important role in disaster response (Table 2) since the majority of people would search for safety shelters in monasteries and other religious buildings (85.0%). Also, public buildings such as schools, ward administrative offices, and community halls were mentioned as safe shelters (36.0%).

**Table 2:** List of mentioned safe shelters

Safe shelters	Per cent of cases (%)
Religious buildings	85.0
Public buildings	36.0
Public spaces	20.4
Work/private places	4.9
Others	5.3

The survey also collected ideas for voluntary activities. They ranged from a general support in form of cooperation with neighbours and administration or donations (63.8%) to more specific ideas for disaster management. Ideas for helping in disaster response were immediate rescue operations, helping those in need, providing/sharing food/water and helping in health care (23%). For disaster rehabilitation, people would support rebuilding and social affairs (5.7%). Asked which activities could assist disaster preparedness, respondents mentioned ideas to clean drainages and potential disaster areas and to warn people and increase disaster risk awareness (12%) (Fig. 3h).

## Discussion

This study provides a basis for further research into a general framework of disaster preparedness at the individual/household level. It does so by permitting implications about the personal resilience and private disaster preparedness in Yangon to be derived. In general, Yangon’s civil society can cover the first 72 h in disastrous events since its stockpiling behaviour concerning water and food is in accordance with the recommendations of YCDC and the SPHERE project (Sphere Association 2018; YCDC 2020). Their suggested standards are based on the Humanitarian Charter, and they have received broad acceptance in humanitarian disaster response and the preparedness sector. According to the SPHERE guideline, the minimum amount of drinking water needed per day per person is 2.5–3 L (Sphere Association 2018). The YCDC recommends stockpiling water bottles and (dry) food for three days per household in order to be prepared for an eventual evacuation (YCDC 2020). However, these amounts are based on experts’ estimations and publications from foreign countries, and knowledge is needed about local standards of daily consumption and stockpiling behaviour.

With an average storage of eight litres of drinking water per person in the investigated townships, Yangon’s citizens can, theoretically, bridge a maximum of 72 h without access to further drinking water resources. This amount is in-line with the buying frequency of water barrels in the townships, which can be taken for a quick estimation of drinking water availability in households. However, the wide range in the amounts of stored drinking water shows that highly vulnerable households exist in Yangon. Especially in informal or temporal urban settlements in megacities of developing countries, the minimum standards of water, food, and housing are often not met even in regular times as, e.g. a study from Nairobi, Kenya, shows (Patel and Chadhuri 2019). Thus, meeting the provided standards of supply depends on small-scaled urban contexts. Our results show that many people rely on locally based urban water supply systems. In Pazundaung, the average buying frequency of the drinking water barrels is a week, but people have a higher buying frequency, e.g. every two or three days, in other townships. This difference is probably related to the greater number of well-educated and employed inhabitants in Pazundaung, who can afford to stockpile due to better income and potentially have more storage space. To increase the resilience of the general public, these supply systems need to be assessed and analysed to set clear priorities in case of interruptions. Based on the high buying frequency of drinking water in Yangon, drinking water supply chains thus need to be prioritized during disasters.

Yangon’s population seems to be well prepared for short- and medium-term food supply insufficiencies, as more than 70% store enough dry food, such as rice or flour, for one week or longer and alternative cooking devices exist. This result exceeds numbers from, e.g. the Philippines and Thailand, where 50% and only 14.4% of the population stockpile food (Hoffmann and Muttarak 2017), as well as from Hong Kong, where 57.3% store food (Tam et al. 2018).

Alternative cooking devices without electricity are still present in Yangon (Zin Nwe Myint 2006) and in use, as in many developing countries. Areas with frequent power cuts might be better prepared due to a greater flexibility to adapt to alternative forms of cooking. In case of electricity supply disruptions, the households have alternative sources of lighting. However, the results also show that living conditions and behaviour in urban areas reduce the capacity for coping with food supply insufficiencies. Due to the high and immediate availability of food, shops, and goods in regular times, some people see no need for extra stockpiling. It also might not be common to have extra food at home as, especially in the densely settled areas of Yangon, living space is limited and consuming street food daily is common. Urban areas are characterized by a higher number of single households and workers and business households, which are highly vulnerable during disasters due to deficient social embedding and urban anonymity.

Resilient societies are able to flexibly respond to or cope with failures of critical infrastructure and to adapt and learn to keep societal life running (Birkmann et al. 2016; Solecki et al. 2017; United Nations 2020). This response includes private stockpiling of minimum supplies of water, food, communication facilities, etc. as mentioned above. It also covers private alternatives of coping with interruptions of electric power supply and the individuals’ access to official news and information. What furthermore matters are stockpiling of medication and first-aid equipment and knowledge about first-aid techniques in a community. Our results show a wide underestimation of the efficacy of first-aid during and after emergencies or disasters. In Myanmar, learning first-aid techniques is not established in school curricula nor required for getting a driving licence. This implies a poor response capacity to treat injured people.

Our study shows a broad availability of mobile phones in urban areas since almost all of the households have at least one mobile phone. The high availability of mobile phones and power banks potentially make mobile phones and social media good sources for risk communication (Myat Htut Nyunt et al. 2015; Cheng et al. 2016; Lai et al. 2018). The percentage of smart phones users in Yangon is, however, unclear as it was not separately accessed in the survey. It is therefore not clear how many households could access social media, apps, and other sources in case of disasters. However, we assume a high distribution of smart phones and that most of the respondents can access social media and apps. Smart phones could, thus, be used as alternatives to TV and radios when communicating with the general public during disasters. The TV is still the main information source and would reach to the majority of the Yangon population. In comparison to that, in the USA, for instance, in case of emergency only 25% would go for TV for information and 60% would visit specific websites (Kapucu 2008). It shows that the use of the internet as source of information is not yet widely established in Yangon.

General knowledge about natural hazards and the ability to recognise early warning signs of approaching hazards are key factors in individual resilience and anticipative capacities. These aspects can substantially increase survival chances in case of disasters. People affected by cyclone Nargis reported that they did not get proper alerts in advance and thus had been surprised by the cyclone’s intensity (Nyan Win Myint et al. 2011). Initiatives for increasing institutional preparedness started after cyclone Nargis in 2009 (Zin Mar Than et al. 2020). Knowledge and behaviour during disasters differ on small scales between townships and between people with different educational backgrounds in Yangon. These aspects are central factors for risk perception and awareness (Hoffmann and Muttarak 2017). A study of household resilience concerning flooding in Bangladesh, for instance, has shown a positive impact of education on household resilience (Mondal et al. 2021).In our study, we assessed also a tendency to this correlation.

Townships where more experience with disasters was reported, such as Dawbon, might have a better routine in coping by more frequently experiencing and responding to disasters. Thus, previous disaster experience may have a positive impact on anticipation capacity. However, the mentioned coping strategies are not always the most effective or life-saving measures as they more intuitively developed out of a disastrous situation.

For evacuation situations, all mentioned belongings were important, which indicates a certain level of resilience in the general public. The answers suggest that family members stay together and do not lose each other during an evacuation. The majority took for granted leaving their houses with all family members. In case of a natural hazard, chaotic situations may occur and groups of people may be split or individuals can get lost. In order to immediately start joint searches for lost persons with professionals, it is important to have to hand up-to-date pictures from all family and group members. Only 1.4% mentioned their concerns about being separated from their families in case of disasters, while people mainly expressed their concerns about health and life of their family members (36.0%).

During the survey, we observed concerns of peoples on talking about natural hazards for religious or spiritual reasons. People were afraid to probably provoke the occurrence of natural hazards by talking about them or getting bad karma as a consequence. A sensitive disaster management must thus see religion and spiritual/religious institutions separately from disaster prevention and preparedness issues. This perspective might motivate more people to voluntarily become involved in disaster preparedness. Religious buildings play an important role in disaster response as safe shelters because the locations of monasteries and other religious buildings are well known in the neighbourhoods and these locations can be easily communicated. Furthermore, many people associate religion and monasteries and other religious buildings with places of safety and security. The majority of people search for safety shelter in monasteries and other religious buildings (85.0%). When the usual communication systems fail, these shelters could be equipped with alternative communication systems, e.g. radios, and thus serve as information nodes in case of disaster.

There is a high solidarity in the civil society. Potentials and opportunities for volunteer work in different stages of disasters exist; however, they need to be better coordinated and promoted. More offers in voluntary civil engagements in risk preparedness could, firstly, improve disaster response systems, secondly, strengthen the social fabric of the civil society, and thirdly, counteract the increasing anonymity in urban contexts. These measures would, further, help ensure inclusion and leaving no one behind.

The study shows that megacities, like Yangon, generally have good prerequisites for resilience during and after disasters. However, there is a lack of knowledge on natural hazards and important preparedness measurements for staying capable of acting during and after disasters as well as keeping an acceptable level of stability and prospects for sustainable developments. Comprehensive education of the urban population on specific natural hazards and on household preparedness, such as first-aid techniques could boost the anticipating, preventing and adapting capacities and thus contribute tremendously to strengthening the resilience in urban areas. This study, however, can also provide qualitative results and lacks clear quantifications of urban resilience. More research needs to be carried out on quantifying concepts of resilience and making urban areas’ resilience more comparable.

## Conclusion

In times of increasing urbanization and progressive climate change, and in the presence of the pandemic, societal resilience is more important than ever. Since the beginning of 2020, administrations and governments, societies, citizens, and neighbourhoods worldwide are running in crises management mode due to the CoViD-19 pandemic. Common non-pharmaceutical medium-term intervention measures such as social distancing and lockdowns are forcing economies and daily life to stand still. Especially with the CoViD-19 pandemic and the recent challenges in Myanmar, and the ever-present danger of a disaster due natural hazards (e.g. cyclones, floods, earthquakes), the overall system is severely challenged and already under stress. More than ever, the citizens must rely on their absorptive resilience capacities.

On basis of our study, we can recommend the following to improve societal resilience and private disaster preparedness at the local level in Yangon:

- The immense lack of first-aid knowledge and its ability to increase resilience needs to be addressed. The learning of first-aid-techniques and preparation of first-aid kits should be obligatory in schools at lower and higher grades. Every household should be equipped with a first-aid kit including instructions on individual preparedness, such as a sheet with spaces for relevant phone numbers. Those measures could rescue numerous lives in an emergency and increase the resilience and well-being of many people in Yangon. This is in-line with SDG 3 and the goals of the Sendai Framework 2015 for Disaster Risk Reduction.
- There is an urgent need in all townships for broad information campaigns and community-based methods for disaster preparedness to increase knowledge and resilience in the general public. In-line with this, risk communication via mobile phone needs to be extended and improved since communicating risk awareness, preparedness and response via mobile phone has great potential. Risk and crisis communication needs to be improved from the institutional side to ensure that reliable information from official authorities is also correctly cited in social media. Therefore, mobile phone infrastructure should be strengthened in order to maintain functionality and increased reliability in case of disaster. In parallel, a redundant communication structure, e.g. in central shelters, should be established to ensure emergency calls and important communication in case of a mobile phone network failure.
- There is a high motivation to volunteer as part of disaster management. More opportunities for and better coordination of volunteer work would strengthen civil society and ensure a better social embeddedness of those who run a risk of becoming left behind.
- Disaster preparedness and exchange about potential disasters is perceived as a religiously sensitive topic in Myanmar. Religious facilities need to be prepared for disasters but not necessarily involved in volunteer acquisition. Religious buildings have a key role in disaster response as safe shelters for the general public. Thus, responsible leaders and staff in religious institutions should be trained in first aid and in religious emergency care and emergency psychology. Official buildings are also safe shelters, thus governmental staff and teachers should have at least basic knowledge in emergency psychology. In case the usual communication infrastructure fails, these shelters could be equipped with alternative means of communication (e.g. radios) and function as information hubs to ensure emergency calls and official information.

Urban lifestyles and the normal urban infrastructure reduce resilience through, e.g. a higher buying frequency and availability of daily goods in regular times or small apartments sizes with less space for stockpiling. People see no need and no space to stockpile water and food in regular times. In addition, a high number of single and small households and shared apartments of workers with lower social embeddedness do not stockpile water or food. This development needs to be catered for in the course of (mega) urbanization developments.

Assessing this information in advance of a potential disaster is an essential part of disaster preparedness. It can improve both estimation of the local impacts of a natural hazard and priority-setting in self-preparedness and disaster response. Our findings from Yangon emphasize the general importance of contextualizing disaster impacts and improving the institutional and private disaster responses. They can be used as a basis for future research in other natural disaster-prone regions and (mega) cities, which could lead towards developing a more generalized framework for improving disaster resilience and preparedness.

## Data Availability

The questionnaire is available online as supplementary material.

## Footnotes

1 Some respondents mentioned more than one specific important document.
